# Influence of strength of motivation for medical studies among foreign students

**DOI:** 10.1192/j.eurpsy.2025.1285

**Published:** 2025-08-26

**Authors:** N. Bouattour, F. Guermazi, R. Jbir, F. Cherif, M. Lalaoui Rhali, R. Masmoudi, J. Masmoudi

**Affiliations:** 1 Psychiatry A, Hedi Chaker University Hospital, Sfax, Tunisia

## Abstract

**Introduction:**

The process of successfully joining medical schools, represents an important event for each student globally. The transition from one year to another needs much effort, sacrifice and perseverance in order to obtain the final goal of being a practitioner.

**Objectives:**

To assess the strength of motivation for medical studies in foreign students.

**Methods:**

We conducted a cross-sectional, descriptive and analytical study, carried out between July and September 2023 among foreign students at the Sfax Faculty of Medicine. Data collection was carried out using an anonymous self-questionnaire via “Google Forms shared among students via social media. We used the strength of motivation for medical school “SMMS” in its French validated version.

**Results:**

Seventy-two foreign medical students completed the survey. The average age was 25 ± 3.45 years. Males represented 57% of the total participants. The majority of respondents were single (77.8%). Sixty-one percent were enrolled in the third cycle of medical studies, 26.5% were enrolled in the second cycle of medical studies and 12.5% were enrolled in the first cycle of medical studies. Of those surveyed, 83.3% had chosen medical studies as their first choice. For 59.7% of students, this choice was their own. The mean score for the motivational strength questionnaire was 42.4, as for the sacrifice dimension 14.6, for the start-up readiness 13.7, and 11.02 for the perseverance dimension. The mean score for the strength of motivation for medical studies was significantly lower in students enrolled in second cycle of medical studies (p=0.043) and in those who did not have their own choice of medical studies (p=0.037). We found no statistical association between the strength of motivation and having a family member in the medical field.

**Conclusions:**

Motivation is a crucial aspect of the academic life of international medical students, shaping their learning path and overall experience. These students, often driven by dreams of a medical career, face obstacles linked primarily to adapting to a new educational system. The motivating strength that drives them to embark on and pursue medical training may be fueled by the desire to acquire clinical skills that will contribute to the provision of healthcare in their home countries.

**Disclosure of Interest:**

None Declared

